# Supplementary material for: Effects of physical activity on anxiety levels in college students: mediating role of emotion regulation
Source: PeerJ. 2024 Sep 18;12:e17961. doi: 10.7717/peerj.17961 (PMC11416097; doi:10.7717/peerj.17961)
Supplement: Supplemental Information 3 [file peerj-12-17961-s003.docx]

**Physical exercise**

PE：Physical exercise

TPE：Time for physical exercise

FPE：Frequency of physical exercise

PEI：Physical exercise intensity

**Scoring method：**This scale examines the amount of physical exercise from three aspects: intensity, time and frequency of physical exercise. Exercise amount = intensity × time × frequency, intensity and frequency from 1 to 5 grades, respectively recorded 1 to 5 points, time from 1 to 5 grades, respectively recorded 0 to 4 points, the highest score is 100 points, the lowest score is 0 points, the evaluation standard of exercise amount: ≤19 is classified as fluctuation; 20 ~ 42 were divided into moderate exercise; ≥ 43 is classified as large exercise,The higher the score, the more exercise.

**Anxiety**

SAS：Anxiety

SAS1：I feel more nervous and anxious than usual （Anxiety）

SAS2：I was scared for no reason （Fear）

SAS3：I tend to get upset or frightened （panic-stricken）

SAS4：I think I might go crazy（Feeling of insanity）

SAS5：I think everything's fine and nothing bed's gonna happen, you know （Premonition of misfortune）

SAS6：My hands and feet were shaking （Trembling hands and feet）

SAS7：I suffer from headaches, neck pains and back pains （Somatic pain）

SAS8：I feel weak and tired easily （Fatigue）

SAS9：I feel calm and easy to sit still （Akathisia）

SAS10：I feel my heart beating fast （Palpitation）

SAS11：I suffer from bouts of dizziness （Giddy）

SAS12：I have a faint attack or feel like I'm going to faint （Syncope）

SAS13：I breathe in and out easily （Pneumodiol）

SAS14：I have numbness and tingling in my hands and feet （Tingling of hands and feet）

SAS15：I suffer from stomachaches and indigestion （Stomach pain or indigestion）

SAS16：often have to pee （Frequency of urination）

SAS17：My hands are often dry and warm （Hyperhidrosis）

SAS18：I blush and get hot. （Flushed face）

SAS19：I fall asleep easily and sleep well all night （Sleep disorder）

SAS20：I had nightmares. （Nightmare）

**Scoring method：**SAS uses a 4-level score to evaluate the frequency of symptoms. The standard is: "1" means no or very little time; "2" means sometimes there is; "3" means yes most of the time; 4 indicates most or all of the time. Out of the 20 items, l5 are stated in negative words and graded in the order of l to 4 above. The remaining 5 items (Nos. 5, 9, 13, 17, 19) marked with * are stated in positive words and are scored in reverse order from 4 to 1. The main statistical indicator of SAS is the total score. Add the scores of each of the 20 items to get a rough score; Multiply the rough score by 1.25 and then take the integral part to get the standard score,The higher the score, the more anxiety symptoms.

**Emotional regulation**

ERQ：Emotional regulation

CR：Cognitive reappraisal

EI：Expression inhibition

CR1：When I want to feel something positive (like happy or joy), I change the way：I think about things（Cognitive reappraisal）

EI1：I don't show my emotions（Expression inhibition）

CR2：When I want to feel less negative emotions (like sadness or anger), I CMP：change my perspective（Cognitive reappraisal）

EI2：When I feel positive emotions, I'm very careful not to let them show（Expression inhibition）

CR3：When faced with a stressful situation, I make myself think about it in a way that helps me stay calm（Cognitive reappraisal）

EI3：The way I control my emotions is by not expressing them（Expression inhibition）

CR4：When I want to feel more positive emotions, I change the way I think about the situation（Cognitive reappraisal）

CR5：I control my emotions by changing the way I think about the situation（Cognitive reappraisal）

EI4：When I feel negative emotions, I make sure I don't show them（Expression inhibition）

CR6：When I want to feel less negative emotions, I change the way I think about the situation（Cognitive reappraisal）

**Scoring methods：**The Emotion Regulation Questionnaire compiled by Gross consists of 10 items and is scored on 7 points. The higher the score, the more frequently emotion regulation strategies are used. The scale includes two dimensions: cognitive reappraisal and expression inhibition. The measurement of cognitive reappraisal dimension consisted of 6 items, and the measurement of expression inhibition dimension consisted of 4 items.

**Gender**

1：boys

2：grils

**Grade**

1：freshman

2：Sophomore

3：junior

4：senior

**Address**

1：Urban

2：Rural
