# Supplementary material for: Effects of physical activity on anxiety levels in college students: mediating role of emotion regulation
Source: PeerJ. 2024 Sep 18;12:e17961. doi: 10.7717/peerj.17961 (PMC11416097; doi:10.7717/peerj.17961)
Supplement: Supplemental Information 4 [file peerj-12-17961-s004.pdf]

Hao *et al. BMC Public Health (2022) 22:2054*
https://doi.org/10.1186/s12889-022-14515-9

BMC Public Health

RESEARCH

Open Access

Relationship between body dissatisfaction, insufficient physical activity, and disordered eating behaviors among university students in southern China

Ming Hao[1*], Yifei Fang[1], Wenjing Yan[2], Junwang Gu[1], Yanbin Hao[1] and Chunmei Wu[1]

Abstract

Background: With an increasing incidence of obesity, the relationship between obesity and body image has become a hot research topic worldwide. From high school to university, young people experience changes in their social environment. University students have a high incidence of eating disorders and insufficient physical activity. The purpose of this study was to explore the relationship between body dissatisfaction, insufficient physical activity, and disordered eating behaviors among university students in southern China.

Methods: In total, 1296 university students aged 18–23 years were recruited for this study. The participants completed anthropometric measurements, the Physical Activity Rating scale-3 (PARS-3), and the Chinese-Dutch Eating Behavior Questionnaire (C-DEBQ). The ideal weight and silhouette were reported by university students using a questionnaire.

Results: Compared with men, young women had a higher level of body dissatisfaction. For men, body mass index (BMI; β = 0.76, $P < 0.01$), physical activity score (β = −0.11, $P < 0.01$), and restrained eating score (β = 0.10, $P < 0.01$) were the significant factors predictive of body dissatisfaction. For women, BMI (β = 0.57, $P < 0.01$), muscle mass (β = 0.12, $P < 0.01$), physical activity score (β = −0.11, $P < 0.01$), and restrained eating score (β = 0.09, $P < 0.01$) were the significant factors predictive of body dissatisfaction.

Conclusions: University students with high body dissatisfaction had lower physical activity scores and higher restrained eating scores. The data presented here highlight the impact of university students' body dissatisfaction on physical activity deficiency and disordered eating behaviors in China.

Keywords: Body dissatisfaction, Exercise habits, Eating behavior, Body image, Southern China

Background

The increasing rates of overweight and obesity have become issues of international concern. In 2016, far more than 1.9 billion adults older than 18 years were overweight, and over 650 million were obese [1]. With the continuous development of China's economy, people are paying more and more attention to the health problems caused by obesity [2]. Furthermore, insufficient physical activity deserves attention. Based on a pooled analysis of

*Correspondence: hm48922200@gmu.edu.cn

[1] School of Public Health and Health Management, Gannan Medical University, University Park, Rongjiang new area, Ganzhou City 341000, Jiangxi Province, China
Full list of author information is available at the end of the article

© The Author(s) 2022. **Open Access** This article is licensed under a Creative Commons Attribution 4.0 International License, which permits use, sharing, adaptation, distribution and reproduction in any medium or format, as long as you give appropriate credit to the original author(s) and the source, provide a link to the Creative Commons licence, and indicate if changes were made. The images or other third party material in this article are included in the article's Creative Commons licence, unless indicated otherwise in a credit line to the material. If material is not included in the article's Creative Commons licence and your intended use is not permitted by statutory regulation or exceeds the permitted use, you will need to obtain permission directly from the copyright holder. To view a copy of this licence, visit http://creativecommons.org/licenses/by/4.0/. The Creative Commons Public Domain Dedication waiver (http://creativecommons.org/publicdomain/zero/1.0/) applies to the data made available in this article, unless otherwise stated in a credit line to the data.

the results of 358 population-based surveys with 1.9 million respondents, more than a quarter of adults worldwide fail to reach the appropriate level of physical activity [3]. Insufficient physical activity has also been reported in China. Based on the summary results of reports on nutrition and chronic diseases among Chinese (2015), the rate of regular physical exercise among Chinese residents aged 20–69 years was only 18.7% in 2013 [4]. In addition to being an important influencing factor of obesity, insufficient physical activity also increases the risk of cardiovascular and cerebrovascular diseases, anxiety, insomnia, and depression [5–7].

Young individuals try to change their body shape, which leads to eating disorder behaviors. According to a report on the disordered eating behaviors among American high school students, 21.8% of girls and 11.2 of boys had engaged in disordered eating behaviors in the past 30 days [8]. Moreover, a survey of 18-year-old university students in the United States showed a 31% prevalence of disordered eating behaviors [9]. Disordered eating behavior also exists among young Chinese adults. A study conducted in China showed that 2.5% of young Chinese displayed at-risk eating attitudes [10]. Those with high body mass index (BMI) are found to have an increased risk of disordered eating behaviors [10, 11]. Moreover, disordered eating behavior may affect people's physical health and mental health condition, such as depression [12, 13].

Body image belongs to a psychosocial construct, and was proposed by Schilder in the 1930s as "the picture of our own body which we form in our own mind" [14]. When there is a difference between the actual body and the idealized body, people are dissatisfied with their body shape. Body dissatisfaction is believed to have a negative impact on an individual's physical and mental health [15]. Body dissatisfaction may lead people to take extreme actions to change their body shape. This includes active vomiting and not eating for extended periods [16, 17]. These extreme behaviors may cause an eating disorder [16, 18]. In addition, high body dissatisfaction is also related to the lack of physical activity habits [19].

University students have a high incidence of eating disorders and insufficient physical activity [20]. From high school to university, young people experience changes in their social environment. They organize their lifestyle independently without parental supervision, and the sudden increase in freedom may make it difficult for them to maintain a healthy lifestyle [21, 22]. Simultaneously, young adults need to adapt to increased independence and academic pressure, making universities a pernicious period for mental health problems [23]. Body dissatisfaction is common among university students [19, 23, 24]. Because the lifestyle of university students has a major effect on the formation of a future healthy lifestyle, it is very important to encourage university students to adopt a healthy lifestyle [25]. However, there are few studies on the relationship between body dissatisfaction, disordered eating behaviors, and physical activity among Chinese university students. The purpose of this study was to explore the relationship between body dissatisfaction, insufficient physical activity, and disordered eating behaviors among Chinese university students.

Methods

Participants

A large university was selected in Ganzou city, Jiangxi province, China. This study recruited the study participants through publicity in students' dormitories and study rooms. As there were very few students younger than 18 years, these students were excluded from the study. This Cross-sectional study surveyed 1296 students (men: 643; women: 653) aged 18–23 years who agreed to participate in this study, conducted from July to December 2021.

Measures

Height was measured using a portable stadiometer (Seca 213, Germany) with 0.1 cm precision. Body weight (0.1 kg precision), fat percentage, and muscle mass (0.1 kg precision) were measured with a body composition instrument (Tanita BC-610, Japan). BMI (kg/m2) was calculated using height and weight. The body measurements were performed by MH who is experienced in experience. These instruments have been widely used in the world.

Body image

Regarding body weight dissatisfaction, the ideal weight of young men and women in units (0.1 kg) was recorded using a questionnaire. Subsequently, the ideal BMI was calculated using height and ideal weights, and body dissatisfaction was calculated by combining the actual BMI and ideal BMI values. The difference between the actual BMI and ideal BMI values was the body dissatisfaction score [26].

The participants were asked to complete a set of sex-appropriate silhouettes [27]. The images of the set of silhouettes were numbered between −7 (fat) and 7 (muscle). Participants were asked to select the ideal silhouette that they most liked to possess and the male and female body figures they considered most attractive.

Physical activity

The Physical Activity Rating Scale 3 (PARS-3) was used to measure physical activity, a 5-item self-report scale covering duration, intensity, and frequency [28]. Rating

of each item on a scale of 1 to 5 and the total score for physical activity (i.e., exercise volume) were computed using the following equation: intensity × (duration − 1) × frequency. The range of the total physical activity score was from 0 to 100. According to the total score, physical activity in this study was categorized into four levels: none, total score ≤ 4; low exercise, total score 5–19; medium exercise, total score 20–42; and high exercise, total score ≥ 43.

Eating behavior

The Chinese-Dutch Eating Behavior Questionnaire C-DEBQ [29] was used to assess young people's emotional, external, and restrained overeating style tendencies. Thirteen questions were set for emotional eating, such as "Do you have the desire to eat when you are irritated?"; ten questions were set for external eating, such as "Do you eat more than usual when you see others eating?"; and ten questions were set for restrained eating, such as "Do you find it hard to resist eating delicious foods?". The 5-point Likert scale is applied to the C-DEBQ, with scores ranging from 1 (never) to 5 (always) from low to high. A higher score indicates a higher tendency for the specific type of overeating.

Statistical analyses

An independent t-test was performed to examine the differences in the participants' mean body composition, ideal BMI, body dissatisfaction score, physical activity score, and restrained eating scores by sex. An independent t-test was also used to examine the differences between the silhouette form scores of the most attractive silhouette from the same sex and that from the opposite sex. The Pearson test was used to compare differences between men and women in the BMI and activity level categories. Tukey's test was performed to examine the differences between the mean values of the three different body types of students' total scores for physical activity and restrained eating scores according to sex. Body weight dissatisfaction was used as a dependent variable in multiple regression analysis, whereas muscle mass, BMI, total physical activity score, and restrained eating score were included as predictor variables. The variables were chosen according to the stepwise increase-and-decrease method and a threshold p-value of 0.20, which was calculated using the likelihood ratio test. All parametric tests were conducted on the premise that the data followed a normal distribution. Statistical significance was set at $P < 0.05$. JMP version 16.0 J (SAS Institute Inc., Cary, NC, USA) was used for all statistical analyses.

Results

The participants' characteristics are listed in Table 1. The participants' ages ranged from 18 to 23 years with a mean (± SD) age of 18.7 ± 1.0 years. Among the students, 19.2% were overweight or obese. The overweight and obesity rates of men were higher than those of women ($P < 0.05$), and 29 percentage of participants were classified as not exercising (Table 1). Nevertheless, the medium and high exercise percentages were 15 and 11%, respectively. The mean values of BMI, ideal BMI, fat percentage, muscle mass, and physical activity in men were higher than those in women (Table 1). The average physical activity score of men was 20.8 (Min: 0; Max: 100), higher than that of women, which was 12.3 (Min: 0; Max: 100) ($P < 0.05$). The scores of each dietary behaviors item were higher for women than for men. The average score of women's emotional eating was 26.2 (Min:13; Max:65), higher than that of men's, which was 22.4 (Min: 13; Max: 55) ($P < 0.05$). The average score of women's external eating was 36.4 (Min: 11; Max: 50), higher than that of men's, which was 32.0 (Min: 11; Max: 50) ($P < 0.05$). The average score of women's emotional eating was 28.7 (Min: 11; Max: 50), higher than that of men's, which was 24.8 (Min: 10; Max: 49) ($P < 0.05$).

Table 1 Sample characteristics ($n = 1296$)

	Mean ± SD or n (%)		P
	**Male ($n = 643$)**	**Female ($n = 653$)**	
BMI (kg/m^2)	22.1 ± 3.7	21.2 ± 3.1	< 0.01
Fat%	16.1 ± 6.7	27.7 ± 6.0	< 0.01
Muscle mass (g)	50.4 ± 7.6	36.0 ± 4.0	< 0.01
Ideal BMI (kg/m^2)	21.2 ± 2.2	19.3 ± 2.4	< 0.01
Body dissatisfaction(kg/m^2)	0.8 ± 3.5	2.0 ± 2.9	< 0.01
BMI category			
Underweight	95 (15)	106 (16)	< 0.01
Normal	390 (60)	458 (70)	
Overweight & Obesity	158 (25)	89 (14)	
Physical activity score	20.8 ± 20.4	12.3 ± 16.1	< 0.01
Activity level category			
No exercise	138 (21)	242 (37)	< 0.01
Low exercise	256 (40)	307 (47)	
Medium exercise	141 (22)	59 (9)	
High exercise	108 (16)	45 (7)	
Eating behavior			
Emotional eating score	22.4 ± 9.4	26.2 ± 9.8	< 0.01
External eating score	32.0 ± 8.0	36.4 ± 7.1	< 0.01
Restrained eating score	24.8 ± 8.0	28.7 ± 7.3	< 0.01

BMI body mass index. The significance of differences between male and female students was determined by t-test (for quantitative variables) or by Pearson analyses (qualitative variables)

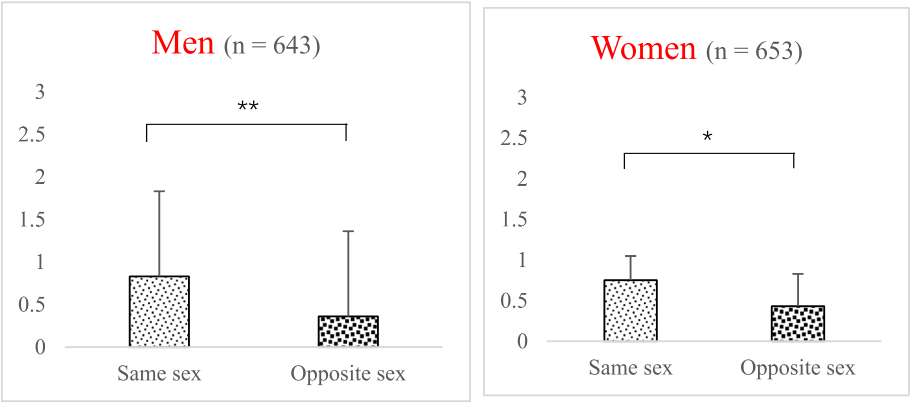

Fig. 1 Ideal silhouette of the same sex and the opposite sex students. * t-test, $P<0.05$; ** t-test, $P<0.01$

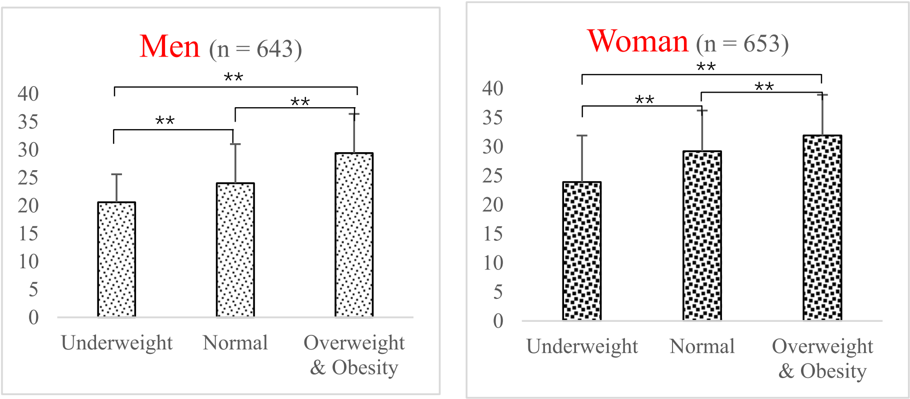

Fig. 2 Restrained eating score of university students with different BMI category. ** Tukey, $P<0.01$

From the male perspective, the most attractive figure of women was thinner than the most attractive figure of men, as shown in Fig. 1. From the female perspective, the most attractive figure of men was thinner than the most attractive figure of women, as shown in Fig. 1.

The restrained eating scores of the students in different BMI categories are shown in Fig. 2. For both men and women, the restrained eating score increased with an increase in the BMI.

Table 2 shown the results for multiple regression analysis of the factors contributing to body dissatisfaction for each sex. For men, BMI (β = 0.76, $P < 0.01$), physical activity score (β = −0.11, $P < 0.01$), and restrained eating score (β = 0.10, $P < 0.01$) were the significant factors predictive of body dissatisfaction. For women, BMI (β = 0.57, $P < 0.01$), muscle mass (β = 0.12, $P < 0.01$), physical activity score (β = −0.11, $P < 0.01$), and restrained eating score (β = 0.09, $P < 0.01$) were the significant factors predictive of body dissatisfaction.

Table 2 Factors that contributed to body dissatisfaction among university students

	β	*t*	VIF	*P*
Men ‡				
BMI (kg/m2)	0.76	31.28	1.22	< 0.01
Physical activity score	−0.11	−5.12	1.04	< 0.01
Restrained eating score	0.10	4.21	1.24	< 0.01
Emotional eating score	−0.04	−1.97	1.06	NS
Women#				
BMI (kg/m2)	0.57	16.80	1.47	< 0.01
Muscle mass (g)	0.12	3.72	1.41	< 0.01
Physical activity score	−0.11	−3.76	1.02	< 0.01
Restrained eating score	0.09	2.82	1.01	< 0.01
Emotional eating score	0.05	1.82	1.02	NS

BMI body mass index, *VIF* variance inflation factor

‡ R^2: 0.69; $P<0.01$; Root Mean Square Error (RMSE): 1.93

R^2: 0.49; $P<0.01$; RMSE: 2.06

Discussion

The results of this study showed that the mean BMI was higher for men than for women; however, the level of body dissatisfaction was higher among women than among men (Table 1). These findings replicate previous research findings [30, 31]. With the constant advertising in the media in the Western countries and even in China, the belief that a woman's thinness is beautiful is deeply rooted in the public's minds [32]. In today's modern society, a slim and graceful figure is considered one of the conditions for women to get more benefits [33]. According to a pioneering study that examined the body image of men and women, women have a higher requirement for a slim body than men; thus, more women try to lose weight than men [34, 35]. Furthermore, behaviors in relation to body monitoring and body change among women in the Chinese society are more pronounced [36]. In this study, a higher level of dissatisfaction with women's bodies was presumed to reflect the socio-cultural background described above.

Studies have shown that men and women have different reasons for body dissatisfaction [32, 37, 38]. Whereas men want a strong body [38], women recognize that their slim figure is considered beautiful [32]. However, the current study found that the higher the body dissatisfaction level of young women, the higher the ideal silhouette scores (Table 2). This shows that young women are satisfied with having a slim body and with the pursuit of muscle acquisition [39]. A study of 388 women aged 17–35 years in Australia also found a change in women's ideal bodies [40]. However, a muscular body requires exercise for a long period, which is more difficult to achieve than that required for a slim body [39]. Therefore, the pursuit of muscle is not unique to men and an important factor affecting women's body dissatisfaction.

Research shows that in the desire for a slim figure, peer perceptions play an important role in body dissatisfaction [41]. With respect to the desire for muscle among both men and women, the ideal silhouette for young people of the opposite sex was thinner than that for young people of the same sex (Fig. 1). The desire for muscles may be related to self-perception. Interestingly, we found that body dissatisfaction among young men was also related to their ideal silhouette of the opposite sex. Well-educated women in Asia are increasingly pursuing education and career opportunities instead of marriage and motherhood [42]. Moreover, the male to female sex ratio in China has risen from 1 to 1.18 [43]. The declining dominance of men in marriage and relationships may be an important reason that men's ideal heterosexual silhouettes are slimmer and their body dissatisfaction is higher.

Moderate-intensity and high-intensity physical activity is recommended by the World Health Organization to reduce the potential risk of chronic disease [6]. The results of this study showed that 29 percentage of young people lacked physical activity habits and only 43% of young people had low physical activity. A lack of physical activity habits among young people in southern China is common. A survey of 650 university students in Guangzhou also found that 28% of young people lacked physical activity habits and 45% of young people were reported to often engage in low physical activity [28]. Exercise intervention is the most direct way to improve low exercise levels in young people. However, simple exercise interventions lack long-term effects on the increase in the exercise level [44].

This study proposes psychological motivations that may aid in improving young people's physical activity levels (Table 2). The results showed that both men and women had higher levels of body dissatisfaction and lower physical activity scores (Table 2). Currently, it is debatable whether body dissatisfaction results in resistance to exercise or motivates participation in physical activity. For example, a survey of 1044 university students from 17 countries on body dissatisfaction and physical activity relationships supports that body dissatisfaction is an important motivation for young people to participate in sports [45]. However, compared to participating in physical activity for health, participating in physical activity to improve appearance is not sustainable [19]. Concomitantly, body dissatisfaction leads to the avoidance of physical activity [19]. For example, people with a higher level of body dissatisfaction are more likely to think that physical activity is embarrassing and they will avoid sports and activities involving motor skills to avoid being considered unattractive [46]. Therefore, reducing body dissatisfaction and cultivating health awareness are of great significance for improving the current situation of low physical activity among young people.

The higher the body dissatisfaction level among university students, the higher their restrained eating score (Table 2). A study of more than 18,500 university students from 22 countries showed that both men and women in Asian countries were more likely than those in other regions to try to lose weight, indicating that Asian university students had a high level of body dissatisfaction [47]. Body dissatisfaction is considered one of the important reasons for eating disorders [48]. Like other Asian countries, Chinese university students also have body dissatisfaction [49] and may lead to dieting (Table 2). The situation of obese university students may be more prominent. To have a slim body, overweight women are more likely to perform restricted fasting or skip meals and engage in dietary restriction behaviors than their normal-weight peers [50]. A retrospective study of 179 young people aged 12–22 years in the

United States found that adolescents with a history of overweight or obesity accounted for a large proportion of people who controlled their weight through dietary restrictions [51]. The results of this study support those of previous studies. Young people with obesity scored higher on restricted eating. Obese young people are more likely to have eating disorders because others do not believe that they are really dieting, and they may even be praised for unhealthy eating behaviors [52]. With the continuous increase in obesity in China and worldwide, the problem of dietary restriction may become more serious in the future. To prevent obesity, sustainable healthy eating and physical activity patterns rather than dieting should be encouraged.

The results of this study emphasized the relationship between body dissatisfaction, BMI, and food restrictions in young people. The higher the level of body dissatisfaction, the higher the restrained eating score, which may aggravate obesity. Restricted eating may negatively affect obesity. First, the effect of restricted eating on body improvement is usually short-term [44]. Second, the compensatory relationship between restrictive behavior and overeating leads to obesity and other diseases [21]. Therefore, establishing a positive body image will help prevent and improve disordered eating in young people. Studies have proven that eating disorders can be improved effectively by body dissatisfaction interventions. Moreover, the effect of improving body dissatisfaction is better than that of direct intervention in dietary behavior [12]. The findings of this study, combined with the results of other previous studies [52], clarify the association between body dissatisfaction and eating disorders and emphasize the importance of improving body dissatisfaction in young people.

Limitations

This study had some limitations. First, the small sample size in this study included only young people aged 18–23 years. Moreover, as this was a cross-sectional study, causal inferences should not be made. In addition, only southern China was surveyed in this study. Therefore, future work should address body image in northern China for comparison.

Conclusions

Our findings indicated that young women had a higher level of body dissatisfaction than men. The data presented here highlight the impact of university students' body dissatisfaction in China on physical activity deficiency and disordered eating behaviors.

Abbreviations

BMI: Body mass index; C-DEBQ: Chinese-Dutch Eating Behavior Questionnaire; PARS-3: Physical Activity Rating Scale 3.

Acknowledgments

We would like to express our appreciation to all the study participants.

Authors' contributions

Study design: M.H., Y.F, W.Y., and C.W. Data collection: M.H., Y. F, W.Y., J. G., Y. H., and C.W. Data analysis: M.H. and Y.F. Manuscript writing: M.H., Y.F., and W.Y. The author(s) read and approved the final manuscript.

Funding

This study was supported by the Starting Research Fund from the Gannan Medical University.

Availability of data and materials

The datasets used and analyzed during the current study are available from the corresponding author on reasonable request.

Declarations

Ethics approval and consent to participate

This study was approved by the ethics committee of the Gannan Medical University, China, No: 2021110. This study was conducted according to the guidelines in the Declaration of Helsinki, and all study participants provided informed consent, agreeing to the required measurement and survey completion procedures. All methods were performed in accordance with the relevant guidelines and regulations.

Consent for publication

Not applicable.

Competing interests

The authors declare no conflict of interest.

Author details

[1]School of Public Health and Health Management, Gannan Medical University, University Park, Rongjiang new area, Ganzhou City 341000, Jiangxi Province, China. [2]Key Laboratory of Prevention and Treatment of Cardiovascular and Cerebrovascular Diseases, Ministry of Education, Gannan Medical University, Ganzhou 341000, Jiangxi, China.

Received: 17 May 2022 Accepted: 1 November 2022
Published online: 09 November 2022

References

1. WHO. Obesity and overweight. 2021. http://www.who.int/news-room/fact-sheets/detail/obesity-and-overweight/. Accessed 2 May 2022.
2. Wang Y, Zhao L, Gao L, Pan A, Xue H. Health policy and public health implications of obesity in China. Lancet Diabetes Endo. 2021;9:446–61.
3. Guthold R, Stevens GA, Riley LM, Bull FC. Worldwide trends in insufficient physical activity from 2001 to 2016: a pooled analysis of 358 population-based surveys with 1·9 million participants. Lancet Glob Heal. 2018;6:e1077–86.
4. National Health Commission of the P.R. China. Report on nutrition and chronic diseases of Chinese residents. 1rd ed. Beijing: People' Medical Publishing House; 2015. (in Chinese)
5. WHO. The world health report 2007: a safer future global public health security in the 21ST century: Global Public Health; 2007.
6. WHO. Global recommendations on physical activity for health WHO. Geneva: World Health Organization; 2010.
7. Tian Y, Jiang C, Wang M, Cai R, Zhang Y, He Z, et al. BMI, leisure-time physical activity, and physical fitness in adults in China: results from a series of national surveys, 2000-14. Lancet Diabetes Endo. 2016;4:487–97.

8. Pisetsky EM, Chao YM, Dierker LC, May AM, Striegel-Moore RH. Disordered eating and substance use in high-school students: results from the youth risk behavior surveillance system. Int J Eat Disord. 2008;4:464–70.
9. Barrack MT, West J, Christopher M, Pham-Vera AM. Disordered eating among a diverse sample of first-year college students. J Am Coll Nutr. 2019;38:141–8.
10. Liao Y, Knoesen NP, Castle DJ, Tang J, Deng Y, Bookun R, et al. Symptoms of disordered eating, body shape, and mood concerns in male and female Chinese medical students. Compr Psychiatry. 2010;51:516–23.
11. Al Banna MH, Brazendale K, Khan MSI, Sayeed A, Hasan MT, Kundu S. Association of overweight and obesity with the risk of disordered eating attitudes and behaviors among Bangladeshi university students. Eat Behav. 2021;40:101474.
12. Gao HQ, Wang BX, Sun LL, Li T, Wu L, Fu LG, et al. The mediating effect of body dissatisfaction in association between obesity and dietary behavior changes for weight loss in Chinese children. Biomed Environ Sci. 2019;32:639–46.
13. Khraisat BR, Al-Jeady AM, Alqatawneh DA, Toubasi AA, AlRyalat SA. The prevalence of mental health outcomes among eating disorder patients during the COVID-19 pandemic: a meta-analysis. Clin Nutr ESPEN. 2022;48:141–7.
14. Schilder PM. The image and appearance of the human body: studies in the constructive energies of the psyche. New York: International Universities Press; 1978.
15. Mellor D, Waterhouse M, Mamat NH, Xu X, Cochrane J, McCabe M, et al. Which body features are associated with female adolescents' body dissatisfaction? A cross-cultural study in Australia, China and Malaysia. Body Image. 2013;10:54–61.
16. Forman-Hoffman V. High prevalence of abnormal eating and weight control practices among US high-school students. Eat Behav. 2004;5:325–36.
17. Muris P, Meesters C, Blom W, Mayer B. Biological, psychological, and sociocultural correlates of body change strategies and eating problems in adolescent boys and girls. Eat Behav. 2005;6:11–22.
18. Hayes JF, Fitzsimmons-Craft EE, Karam AM, Jakubiak J, Brown ML, Wilfley DE. Disordered eating attitudes and behaviors in youth with overweight and obesity: implications for treatment. Curr Obes Rep. 2018;7:235–46.
19. More KR, Phillips LA, Eisenberg Colman MH. Evaluating the potential roles of body dissatisfaction in exercise avoidance. Body Image. 2019;28:110–4.
20. Sandoz EK, Boullion GQ, Mallik D, Hebert ER. Relative associations of body image avoidance constructs with eating disorder pathology in a large college student sample. Body Image. 2020;34:242–8.
21. Forestell CA, Spaeth AM, Kane SA. To eat or not to eat red meat. A closer look at the relationship between restrained eating and vegetarianism in college females. Appetite. 2012;58:319–25.
22. Muñoz-Rodríguez JR, Luna-Castro J, Ballesteros-Yáñez I, Pérez-Ortiz JM, Gómez-Romero FJ, Redondo-Calvo FJ, et al. Influence of biomedical education on health and eating habits of university students in Spain. Nutrition. 2021;86:111181.
23. Uri RC, Wu Y-K, Baker JH, Munn-Chernoff MA. Eating disorder symptoms in Asian American college students. Eat Behav. 2021;40:101458.
24. Lee T-C, Chiang C-H, Chu C-L. Body image promotion program with a creative movement approach for female college students in Taiwan. Mental Health Prevent. 2019;13:107–12.
25. Ramírez-Contreras C, Farrán-Codina A, Izquierdo-Pulido M, Zerón-Rugerio MF. A higher dietary restraint is associated with higher BMI: a cross-sectional study in college students. Physiol Behav. 2021;240:113536.
26. Arroyo M, Basabe N, Serrano L, Sanchez C, Ansotegui L, Rocandio AM. Prevalence and magnitude of body weight and image dissatisfaction among women in dietetics majors. Arch Latinoamer. 2021;60:126–32.
27. Frederick DA, Buchanan GM, Sadehgi-Azar L, Peplau LA, Haselton MG, Berezovskaya A, et al. Desiring the muscular ideal: Men's body satisfaction in the United States, Ukraine, and Ghana. Psychol Men Masculinity. 2007;8:103–17.
28. Yang G, Li Y, Liu S, Liu C, Jia C, Wang S. Physical activity influences the mobile phone addiction among Chinese undergraduates: the moderating effect of exercise type. J Behav Addict. 2021;10:799–810.
29. Wang Y-F, Ha S, Zauszniewski JA, Ross R. Psychometric properties of the Chinese version of the Dutch eating behavior questionnaire in a sample of Taiwanese parents. Obes Res Clin Pract. 2018;12:129–32.
30. Chen H, Jackson T. Are cognitive biases associated with body image concerns similar between cultures? Body Image. 2005;2:177–86.
31. Mellor D, McCabe M, Ricciardelli L, Merino ME. Body dissatisfaction and body change behaviors in Chile: the role of sociocultural factors. Body Image. 2008;5:205–15.
32. Xu X, Mellor D, Kiehne M, Ricciardelli LA, McCabe MP, Xu Y. Body dissatisfaction, engagement in body change behaviors and sociocultural influences on body image among Chinese adolescents. Body Image. 2010;7:156–64.
33. Teng F, You J, Poon K-T, Yang Y, You J, Jiang Y. Materialism predicts young Chinese women's self-objectification and body surveillance. Sex Roles. 2016;76:448–59.
34. Muth JL, Cash TF. Body-image attitudes: what difference does gender make?1. J Appl Soc Psychol. 1997;27:1438–52.
35. Paxton SJ, Wertheim EH, Gibbons K, Szmukler GI, Hillier L, Petrovich JL. Body image satisfaction, dieting beliefs, and weight loss behaviors in adolescent girls and boys. J Youth Adolesce. 1991;20:361–79.
36. Sai A, Othman MY, Wan Zaini WF, Tan CS, Mohamad Norzilan NI, Tomojiri D, et al. Factors affecting body image perceptions of female college students in urban Malaysia. Obes Med. 2018;11:13–9.
37. Tiggemann M, Gardiner M, Slater A. "I would rather be size 10 than have straight A's": a focus group study of adolescent girls' wish to be thinner. J Adolescence. 2000;23:645–59.
38. McCabe M, Ricciardelli L. Parent, peer and media influences on body image and strategies to both increase and decrease body size among adolescent boys and girls. Adolescence. 2001;36:225–40.
39. Damasceno VO, Vianna JM, Novaes JS, Lima JPde, Fernandes HM, Reis VM. Relationship between anthropometric variables and body image dissatisfaction among fitness center users. Rev Psicol Deporte. 2011;20:367–82.
40. Bell HS, Donovan CL, Ramme R. Is athletic really ideal? An examination of the mediating role of body dissatisfaction in predicting disordered eating and compulsive exercise. Eat Behav. 2016;21:24–9.
41. McCabe MP, Ricciardelli LA. A prospective study of pressures from parents, peers, and the media on extreme weight change behaviors among adolescent boys and girls. Behav Res Ther. 2005;43:653–68.
42. You J, Yi X, Chen M. Love, life, and "leftover ladies" in urban China: staying modernly single in patriarchal traditions. China Econ Rev. 2021;68:101626.
43. Nie G. Marriage squeeze, marriage age and the household savings rate in China. J Dev Econ. 2020;147:102558.
44. Hao M, Han W, Yamauchi T. Short-term and long-term effects of a combined intervention of rope skipping and nutrition education for overweight children in Northeast China. Asia Pac J Public He. 2019;31:348–58.
45. Brudzynski LR, Ebben W. Body image as a motivator and barrier to exercise participation. Int J Exerc Sci. 2010;3:14–24.
46. Leary MR. Self-presentational processes in exercise and sport. J Sport Exercise Psy. 1992;14:339–51.
47. Wardle J, Haase AM, Steptoe A. Body image and weight control in young adults: international comparisons in university students from 22 countries. Int J Obesity. 2006;30:644–51.
48. Arigo D, Schumacher L, Martin M. Upward appearance comparison and the development of eating pathology in college women. Int J Eat Disorder. 2014;47:467–70.
49. Chen H, Gao X, Jackson T. Predictive models for understanding body dissatisfaction among young males and females in China. Behav Res Ther. 2007;45:1345–56.
50. Olvera N, McCarley K, Matthews-Ewald MR, Fisher F, Jones M, Flynn EG. Pathways for disordered eating behaviors in minority girls. J Early Adolesc. 2016;37:367–86.
51. Lebow J, Sim LA, Kransdorf LN. Prevalence of a history of overweight and obesity in adolescents with restrictive eating disorders. J Adolescent Health. 2015;56:19–24.
52. Neumark-Sztainer D. Higher weight status and restrictive eating disorders: an overlooked concern. J Adolescent Health. 2015;56:1–2.

Publisher's Note

Springer Nature remains neutral with regard to jurisdictional claims in published maps and institutional affiliations.
