# Supplementary material for: Effects of physical activity on anxiety levels in college students: mediating role of emotion regulation
Source: PeerJ. 2024 Sep 18;12:e17961. doi: 10.7717/peerj.17961 (PMC11416097; doi:10.7717/peerj.17961)
Supplement: Supplemental Information 5 [file peerj-12-17961-s005.pdf]

AKADÉMIAI KIADÓ

Physical activity influences the mobile phone addiction among Chinese undergraduates: The moderating effect of exercise type

Journal of Behavioral Addictions

10 (2021) 3, 799–810

DOI:
10.1556/2006.2021.00059
© 2021 The Author(s)

FULL-LENGTH REPORT

GUAN YANG[1,2*], YUEXIANG LI[3], SHIJIE LIU[4], CHUANNAN LIU[2], CHEN JIA[2] and SONGTAO WANG[2**]

[1] School of Physical Education, South China University of Technology, Guangzhou, China

[2] School of Physical Education & Sports Science, South China Normal University, Guangzhou, China

[3] Department of Physical Education, Guangzhou Vocational University of Science and Technology, Guangzhou, China

[4] School of Physical Education and Sport Training, Shanghai University of Sport, Shanghai, China

Received: October 20, 2021 • Revised manuscript received: June 25, 2021; August 11, 2021 • Accepted: August 12, 2021
Published online: September 17, 2021

ABSTRACT

Background and aims: Mobile phone addiction (MPA) has recently aroused much attention due to its high incidence and considerable health hazards. Although some existing studies have documented that physical activity is negatively associated with MPA, it is little known about the potential effects underlying this relation. The aim of this study was to investigate the relationship between physical activity and MPA among undergraduates in China, and to further examine the moderating effect of exercise type in the relation between them. *Methods:* By the quota sampling, a total of 650 participants engaged in this survey and completed relevant measurements including physical activity rating scale-3 (PARS-3) and mobile phone addiction tendency scale (MPATS). *Results:* Gender ($\beta = 0.271$, $P < 0.05$) and major ($\beta = -0.169$, $P < 0.05$) could significantly predict MPA, respectively, and physical activity was an imperative protective factor to decrease MPA ($\beta = -0.266$, $P < 0.001$). While the physical activity level enhanced from none exercise to medium exercise, an optimum dose-response relationship would emerge between physical activity and MPA ($F_{(3,604)} = 4.799$, $P < 0.01$). Most important, the relation between physical activity and MPA can be moderated by exercise type. Especially in terms of aerobic endurance exercise, the higher level of physical activity the undergraduates performed, the lower degree of MPA would be suffered by them ($\beta = -0.266$, $P < 0.001$). *Discussion:* These findings could be conducive to better understand the positive and potential effects of physical activity on the intervention in MPA, and served as a persuasive evidence that as for university students, actively engaging in aerobic endurance exercise with the medium activity level would be a practicable exercise strategy to deal with MPA in daily lifestyle behavior.

KEYWORDS

physical activity, mobile phone addiction, exercise type, moderating effect, aerobic endurance exercise, Chinese undergraduates

*Corresponding author. Tel.: +8615626499157.
E-mail: yangmp6@m.scnu.edu.cn

**Corresponding author.
E-mail: songtaowang@126.com

AKJournals

INTRODUCTION

Nowadays, with the rapid development and popularity of the internet technology and artificial intelligence, and widespread utilization of these techniques in mobile communication equipment, the mobile phone, as an important electronic product in daily life, has been increasingly embraced by a large body of individuals all over the world (Liu, Xiao, Yang, & Loprinzi, 2019; Xavier, Chamarro, Ursula, Beatriz, & Mariona, 2018). It is no exaggeration to say that, no matter children, adults, or the aged, they have been deeply captured by a

Unauthenticated | Downloaded 02/19/24 07:20 AM UTC

multitude of convenient applications and functions from mobile phones. In accordance with the 44th statistics report from the China Internet Network Information Center (CNNIC, 2019) by the end of 2019, mobile phone users in China have reached 847 million, and the proportion of employing mobile phones to access the Internet is as high as 99.1%. Moreover, among all mobile phone users, the group aged from 20 to 29 has nearly accounted for 1/4, which has been the largest group of them. In reality, it is hardly arduous to see that the majority of this group may be surmised to be university students in China.

As we all know, as a kind of convenient, immediate, and multifunctional mobile digital communication device, mobile phones have been widely used, especially for undergraduate students (Xiang et al., 2020). They can apply those not only to communicate with others, but also buy and pay online, as well as watching videos and learning relevant course (Liu, Liu, & Wei, 2014; Servick, 2015). Accordingly, there is little doubt that this advanced electronic equipment has totally penetrated into every aspect of undergraduates' daily life. However, it is a pity that a growing number of university students have abandoned themselves to mobile phones up to now. What's worse, excessive mobile phone usage would trigger severe consequences, especially the mobile phone addiction (MPA) (Chóliz, 2010), which could be also named as problematic mobile phone use (Bianchi & Phillips, 2005) or mobile phone dependency (Toda, Monden, Kubo, & Morimoto, 2006). MPA, in fact, is a series of compulsive disorder usage caused by excessive mobile phone use or irrational mobile phone use, and also has a great passive and negative effects on individuals about physical and psychological aspects, along with sociable functions such as interpersonal communication in daily life (Chóliz, 2010; Kim, 2013), which not only resembles problematic internet use but also has closely relationships with each other due to through mobile phones people could easily connect to WiFi and then carry out various kinds of online activities according to their different actual needs (Billieux, 2012). It can be obviously seen that mobile phones are only an indispensable media for online surfing by them.

Generally speaking, individuals addicted to mobile phones may fail to control himself in use of this electronic device, especially in terms of occupying times and time which would go far beyond the normal use compared to the general population. In terms of classification, Billieux, Maurage, Lopez-Fernandez, Kuss, and Griffiths (2015) proposed that although MPA was highly similar to behavioral addiction, but it still lacked of sufficient evidence to support this standpoint especially in several main aspects such as loss of control, tolerance symptom, and withdrawal behaviors. Furthermore, Billieux (2012) also summarized prior work and clearly put forth predictors of problematic use of the mobile phone including socio-demographic factors, personality traits and related psychological mechanisms, and self-esteem and related psychological mechanisms. What's more, a pathway model of problematic mobile phone use has been revealed by him soon afterwards (Billieux et al., 2015). No doubt, all these work would be largely conducive to further disclose and explore MPA in coming days.

It is unfortunate that the preceding research has found that the occurrence ration of mobile phone addiction in China may be from 22% to 32% among university students (Yang, Li, Liu, & Wang, 2020), and Jun (2016) also disclosed that among Korean adolescents, the rate of MPA was from 21.4% to 27.4%. As for undergraduates, it is no denying that mobile phone overuse can give rise to not only a poor academic performance (Leep, Barkley, & Karpinski, 2015; Li, Lepp, & Barkley, 2015), but also a tremendous adverse effect on mental health (Aleksandar et al., 2018; Demirci, Akgönül, & Akpinar, 2015; Haruka, Tomoko, Akiyo, & Hisataka, 2017). Hence, a better understanding of relevant risk factors for MPA to university students and potential effects between physical exercise and MPA would be urgently in need to find effective prevention and intervention efforts in the near future.

Physical activity and mobile phone addiction

Physical activity, as we all know, has been regarded as an indispensable behavior in our daily life for a long time, which can bring extensive healthy benefits for us including reduction of the risk with cardiovascular diseases and certain cancers, the symptoms of anxiety, depression, insomnia, and sociable stress (WHO, 2007 & 2010). Since 2010, the World Health Organization has intensely recommended that the moderate-intensity physical activity with at least 150 min or the vigorous-intensity activity with more than 75 min per week, or an equivalent volume of moderate to vigorous physical activity should be met, in response to decrease quite a few underlying risks of chronic diseases (WHO, 2010). However, the preceding studies have verified that MPA may directly or indirectly disrupt college students' physical activity (Lepp et al., 2013). Similarly, several published research has also shown that an obviously reverse association existed between physical activity and MPA (Kim, Kim, & Jee, 2015; Penglee, Christiana, Battista, & Rosenberg, 2019; Yang et al., 2020).

Barkley and Lepp (2016) argued that mobile phone use evidently increased the likelihood of sedentary behavior, and greatly reduced exercise intensity during physical activity. Actually, both would be vital risk factors to interfere with exercise in college students. Through a cross-sectional survey research, Yang, Tan, Li, Liu, and Wang (2019) also revealed that physical exercise could significantly and negatively predict the mobile phone dependence among university students, but this positive effect was totally controlled by a mediator, namely self-control. In addition, a latest review suggested that inappropriate mobile phone usage was closely related to low levels of motivation and practice of physical activity (Zagalaz-Sánchez, Cachón-Zagalaz, Sánchez-Zafra, & Lara-Sánchez, 2019). Based on the above, it can be seen that some valuable findings have been made so far, but they are still sporadic and inadequate, and the potential influential factors between physical exercise and MPA should be further examined. In particular, the

Unauthenticated | Downloaded 02/19/24 07:20 AM UTC

possible moderating effects underlying this relation has not been explored up till now, which also weakens effective intervention in the MPA of undergraduate students.

The moderating effect of exercise type

Exercise type, as an exceedingly crucial element, is an imperative variable to describe the feature of physical activity, of which the importance can be viewed as equivalent as three other important components (e.g., exercise intensity, exercise duration, and exercise frequency). Actually, they are four decisive factors of physical activity and usually regarded as the principle of FITT, which may exert a tremendous effect on the final effects of physical activity (Rhodes, Janssen, Bredin, Warburton, & Bauman, 2017). Therefore, it is possible to speculate that exercise type would be an important potentially factor to influence the relationship between physical activity and MPA. According to the preceding studies, it can be seen that a negative correlation could be existed between physical exercise and MPA (Xu, 2019; Yang et al., 2019), which means physical activity would be regarded as an effective mean for intervening in MPA. Nevertheless, it is unknown so far that whether the intervention effects on MPA by physical activity can be influenced by different kinds of exercise type. In other words, with the same exercise intensity, duration, and frequency, whether just changing exercise type could effectively reduce the MPA during physical activity.

A few previous work have devoted to examine the actual effect of physical exercise intervening in MPA and also got some substantive results (Fan & Yuan, 2015; Ge, Zhong, & Chen, 2015; K. Wang, 2016; X.T. Wang & Zhang, 2016; Xu, 2019; Zhang, Hu, & Xu, 2016; Zhu, 2017), but the following questions are still unresolved. Firstly, exercise type adopted by the study might be complex and also combine with each other, such as running, taichi, swimming, dancing, yoga and all sorts of ball sport, rather than the single one, so we do not know the specific effect about various sorts of exercise type. Moreover, some of them only examine the effect between the control group and treatment (experimental) group, without reciprocal comparison between experimental groups. Lastly, the probability of potential effects are not sufficiently investigated by them while examining the association of physical activity and MPA. Consequently, only a relatively ambiguous and general conclusion that physical activity would put an affirmative effect on the intervention in MPA can be drawn by us.

Admittedly, the solutions to these problems mentioned above might be useful to confirm whether the exercise type can moderate the correlation between physical activity and MPA, and which one may produce the most satisfactory intervention effect. But so far, little work would like to make an attempt to explore the possible roles of exercise type in the relation between physical activity and MPA.

The present study

The objective of this study was to further explore the relationship between physical activity and MPA, and whether the exercise type can moderate the correlation of them. To be specific, three main aspects were included as follows: physical activity would be negatively associated with MPA; with the increase of physical activity level, the degree of MPA would be decreased; the association between physical activity and MPA would be moderated by exercise type.

METHODS

Participants and procedures

The present study adopted a cross-sectional survey design comprising several self-reported standard scales. Using the quota sampling, nearly 60 to 70 students were selected randomly from each of the ten universities in Guangzhou Higher Education Center, China, and 650 undergraduates were finally collected to participate in this paper-and-pencil test. This survey was anonymous and confidential, as well as all questionnaires were completed in accordance with the voluntary principle. Through the supplementary examination, data of 42 samples were invalid so that the final sample size was 608 subjects, and the overall response rate was 93.53%. Of them, 158 were males (26%) and 450 were females (74%); 317 were from liberal arts majors (52.1%) and 291 from science majors (47.9%); 153 came from rural areas (25.2%) and 455 from urban areas (74.8%). In addition, the mean age of all participants in the current study was (20.06 ± 1.98) years, and every student had at least one internet-accessible mobile phone in daily life.

This study was carried out in accordance with the Declaration of Helsinki, and approved by the Institutional Review Board of the South China Normal University. In addition, it was supported by the Innovation Project of Graduate School of South China Normal University (2018LKXM011, 2019WKXM009). All subjects should give their written informed consent before engaging in this survey, and they also had the right to refuse or terminate participation at any time.

Measures

Physical Activity. Physical activity (PA) was measured using the Physical Activity Rating Scale-3 (PARS-3), which is a 3-item self-reported scale comprising intensity, duration and frequency (Liang & Liu, 1994). Each item is evaluated from 1 to 5, and the total score of physical activity (i.e., exercise volume) is computed by the equation below: intensity $\times$ (duration -1) $\times$ frequency, of which the range is from 0 to 100. A total score that is equal to19 or below will be defined as light, while the score from 20 to 42 is defined as medium, and one that is equal to 43 or above is defined as the high exercise. Furthermore, in accordance with previous experience (Xia, Huang, & Liu, 2018; Yang et al., 2019), the present study divided the light exercise into two levels: none exercise equal to or less than 4, and low exercise of 5–19. Therefore, physical activity in the current study contained four levels, from 1 (none exercise) to 4 (high exercise). In

Unauthenticated | Downloaded 02/19/24 07:20 AM UTC

addition, the Cronbach's α of PARS-3 in the current study was 0.64.

Moreover, the current study used a self-designed item to assess exercise type of undergraduates. According to their answers and referring to previous research (Sheng, Jiang, & Gao, 2018), this study divided all exercise programs into four main types: aerobic endurance exercise (e.g., running, swimming, riding bike, and jogging), separated net exercise (e.g., volleyball, badminton, tennis, and table tennis), confrontation exercise (e.g., basketball, football, taekwondo, and free sparring), and difficulty beauty exercise (e.g., dancing, yoga, taichi, and martial arts). Indeed, university students may have more than one exercise program, but they were required to tell us which one he or she took the most frequent. Then we can assign different exercise programs to the corresponding exercise type.

Mobile Phone Addiction. Mobile Phone Addiction Tendency Scale was applied to estimate the mobile phone addiction (MPA) for undergraduates (Xiong, Zhou, Chen, You, & Zhai, 2012), which is based on Young's Internet Addiction Scale (Young, 1998). The MPATS is a 5-point-Likert scale, consisting of 16 items and 4 dimensions: withdrawal symptoms (WS), salience behavior (SB), social comfort (SC) and mood changes (MC). Each item is rated from 1 (completely disagree) to 5 (completely agree), and the total score will be from 16 to 80, and a higher score may mean a deeper degree of MPA. In accordance with classification criteria reported by Young (1998), a total score of 16–31 is classified as "no mobile phone addiction" (no MPA), the score of 32–56 is classified as "possible mobile phone addiction" (possible MPA), and that one equal to or more than 57 is classified as "mobile phone addiction" (MPA). Therefore, MPA in this study consisted of three groups. The classification criteria have been performed fairly in previous studies (Chen et al., 2016; Mei et al., 2018). The internal consistency coefficient (i.e., Cronbach's α) of MPATS in the current study was 0.90, and the alpha coefficient for four sub-dimensions was from 0.55 to 0.82. In addition, the confirmatory factor analysis showed that the overall fitness was fair, and the specific goodness of fit indexes were as follows: χ^2/df = 4.287, RMSEA = 0.074, SRMR = 0.051, TLI = 0.898, CFI = 0.921, GFI = 0.920, IFI = 0.922.

Demographic variables. Several important demographic information such as gender ("1 = *male*" or "2 = *female*"), major ("1 = *liberal arts*" or "2 = *science*"), residence ("1 = *rural*" or "2 = *urban*"), and age was also asked and recorded with a standard survey form.

Statistical analysis

To conduct all statistical analyses, SPSS 25.0 software was applied to this study. The significance level included four layers: $P < 0.10$, $P < 0.05$, $P < 0.01$, and $P < 0.001$. Continuous variables were shown as mean ± standard deviation (SD), and categorical variables were presented as frequency (n) and percentages (%). At first, Chi-square (χ^2) test was executed to compare differences among three groups of MPA regarding some basic demographic information and exercise conditions. Secondly, one-way ANOVA was run to examine the score of MPA and its four aspects at different levels of physical activity and then multiple comparisons between groups used the method of the least significant difference (LSD). Thirdly, using Pearson's correlation analysis to measure the association between physical activity and MPA. Lastly, using Hayes's (2013) PROCESS macro (Model 1) to test whether the effect of physical activity on MPA was moderated by exercise type. As the moderator was a categorical variable, so the independent and dependent variables (i.e., physical activity and MPA) were standardized in this study. The bootstrapping method produced 95% bias-corrected percentile confidence intervals (CI) with these effects from 5,000 resamples of the data. When the upper-level and lower-level CI did not include zero, the effect was significant (Hayes, 2013). At the same time, a simple slope analysis was conducted to examine the essence of the moderating effect, and functions of physical activity on MPA was also graphed at four different kinds of exercise type.

In addition, this study referred to effect sizes including Partial η^2, Cramer's V, and correlation coefficient r (Cohen, 1988) to estimate the magnitude of significant differences during statistical analysis.

Ethics

This study was performed in accordance with the Declaration of Helsinki and approved by the Institutional Review Board of the South China Normal University in response to the involvement of minimal risk and anonymous survey procedures. In addition, all subjects gave written informed consent before participating in this survey, and they also had the right to refuse or terminate the survey at any time.

RESULTS

Descriptive statistics and comparative analysis

In the present study, mobile phone addiction (MPA) was categorized into three groups, and Table 1 displayed the demographic variables and exercise conditions of the whole sample. The percentage of no MPA, possible MPA, and MPA was 13.65% (n = 83), 78.29% (n = 476), and 8.06% (n = 49), respectively.

Chi-square test revealed that there were no statistically considerable differences in residence and exercise type. However, three groups was almost close to the significant level on gender (χ^2 = 5.087, $P < 0.10$, V = 0.091). In addition, significant difference was found in major (χ^2 = 6.739, $P < 0.05$, V = 0.105) and activity level (χ^2 = 19.471, $P < 0.01$, V = 0.137) among them. On the whole, no matter in demographic variables, or in activity level and exercise type, the frequency and percentages for possible MPA was the highest, and followed in turn by no MPA and MPA. But in confrontation exercise and separated net exercise, the

Unauthenticated | Downloaded 02/19/24 07:20 AM UTC

Table 1. Comparisons of socio-demographic variables, physical activity level, and exercise type between subjects among three groups of MPA (*N* = *608*)

	Total (n = 608)	No MPA (n = 83)	Possible MPA (n = 476)	MPA (n = 49)	χ^2	*V*
Gender						
Male	158 (26.0%)	29 (18.4%)	120 (75.9%)	9 (5.7%)	5.087*	0.091
Female	450 (74.0%)	54 (12.0%)	356 (79.1%)	40 (8.9%)		
Major						
Liberal Arts	317 (52.1%)	35 (11.0%)	250 (78.9%)	32 (10.1%)	6.739**	0.105
Science	291 (47.9%)	48 (16.5%)	226 (77.7%)	17 (5.8%)		
Residence						
Rural	153 (25.2%)	28 (18.3%)	116 (75.8%)	9 (5.9%)	4.599	0.087
Urban	455 (74.8%)	55 (12.1%)	360 (79.1%)	40 (8.8%)		
Activity level						
None exercise	169 (27.8%)	21 (12.4%)	131 (77.5%)	17 (10.1%)	19.471***	0.137
Low exercise	273 (44.9%)	28 (10.3%)	225 (82.4%)	20 (7.3%)		
Medium exercise	91 (15.0%)	24 (26.4%)	58 (63.7%)	9 (9.9%)		
High exercise	75 (12.3%)	10 (13.3%)	62 (82.7%)	3 (4.0%)		
Exercise type						
Aerobic endurance	364 (59.9%)	57 (15.7%)	281 (77.2%)	26 (7.1%)	6.805	0.075
Confrontation	70 (11.5%)	6 (8.6%)	56 (80.0%)	8 (11.4%)		
Separated net	63 (10.4%)	4 (6.3%)	53 (84.1%)	6 (9.5%)		
Difficulty beauty	111 (18.3%)	16 (14.4%)	86 (77.5%)	9 (8.1%)		

Note: MPA, mobile phone addiction; * $P < 0.10$; ** $P < 0.05$; *** $P < 0.01$.

frequency and percentages of MPA were higher than no MPA, respectively.

Furthermore, with regard to physical activity level, nearly 45% participants were reported that they often engaged in low exercise, and the percentages for medium and high exercise were 15.0% and 12.3%, respectively. Nevertheless, participants with the proportion of 27.8% were still located in level of none exercise. The most surprising finding was that over half of these participants (59.9%) reported that they frequently took part in aerobic endurance exercise, and then followed by the difficulty beauty exercise, for which the proportion was 18.3%. Two other exercise types (i.e., confrontation exercise and separated net exercise) were almost equivalent, and accounted for 11.5% and 10.4%, respectively.

One-way analysis of variance

For the sake of further exploring the relationship between physical activity and MPA, one-way ANOVA was applied to examine the score of MPA and its four dimensions at different levels of physical activity, which is displayed in Table 2.

The results indicated that the total score of MPA showed significant difference at four activity levels (F = 4.799, $P < 0.01$, η^2 = 0.023). Likewise, apart from the SB, that was also existed in WB (F = 3.906, $P < 0.01$, η^2 = 0.019), SC (F = 4.914, $P < 0.01$, η^2 = 0.024), and MC (F = 4.665, $P < 0.01$, η^2 = 0.023), respectively. What is interesting about the data in this table is that there was an obvious decrease on the score of MPA and its four dimensions,

Table 2. The score of MPA and its four dimensions at different physical activity level

	WS	SB	SC	MC	MPA
Activity level					
None exercise (n = 169)	18.10 ± 4.24	10.26 ± 3.03	8.40 ± 2.92	7.86 ± 2.41	44.62 ± 10.58
Low exercise (n = 273)	17.82 ± 4.28	9.74 ± 3.19	7.95 ± 2.62	7.62 ± 2.36	43.12 ± 10.32
Medium exercise (n = 91)	16.46 ± 5.11	9.31 ± 3.48	7.22 ± 2.63	6.77 ± 2.48	39.76 ± 11.88
High exercise (n = 75)	16.77 ± 4.06	9.93 ± 2.74	7.37 ± 2.39	7.24 ± 2.22	41.32 ± 10.63
$F_{(3,604)}$	3.906**	2.002	4.914**	4.665**	4.799**
Partial η^2	0.019	0.009	0.024	0.023	0.023
Multiple comparisons	None>Moderate** None>High* Low>Moderate*	None>Moderate*	None>Moderate** None>High** Low>Moderate*	None>Moderate*** Low>Moderate**	None>Moderate*** None>High* Low>Moderate**

Note: MPA, mobile phone addiction; WS, withdrawal symptoms; SB, salience behavior; SC, social comfort; MC, mind changes; LSD, least significant difference. * $P < 0.05$; ** $P < 0.01$; *** $P < 0.001$.

Unauthenticated | Downloaded 02/19/24 07:20 AM UTC

especially from none exercise to medium exercise. However, that did not emerge from medium exercise to high exercise.

In addition, multiple comparisons between groups revealed that, no matter in MPA or in its four facets, the score of medium exercise was considerably less than none exercise, and except for SB, that was also significantly less than low exercise. Moreover, the score of none exercise was significantly more than high exercise in MPA, WS, and SC. Taken together, it was not difficult to find that an optimum dose-response relationship may be existed on physical activity and MPA, and that medium activity level might generate the most effective intervention effect for MPA.

Correlation analysis

As shown in Table 3, physical activity was negatively associated with withdrawal symptoms ($r = -0.113$, $P < 0.01$), social comfort ($r = -0.125$, $P < 0.01$), mood changes ($r = -0.087$, $P < 0.05$), and MPA ($r = -0.109$, $P < 0.01$), respectively, but no significant correlation was observed between physical activity and salience behavior. Moreover, MPA and its four dimensions were all positively correlated with each other ($P < 0.001$), and the correlation coefficients ranged from 0.428 to 0.914. According to the criterion of effect sizes, a low relation between physical activity and MPA implied that the relationship of them may be moderated by some other vital factors.

Analysis of moderating effect

To examine whether the relation between physical activity and MPA was moderated by exercise type, the PROCESS macro (Model 1) was carried out to certify the moderating effect underlying this correlation. Due to exercise type was a categorical variable, the aerobic endurance exercise can be set as the reference group in the model. In addition, as the control variable, gender and major would be also put in this model. The main results were presented in Table 4.

As can be seen from analyses of moderating effect, when controlling for gender and major, physical activity could negatively predict MPA ($\beta = -0.266$, $P < 0.001$), but W1 and W2 can positively predict MPA ($P < 0.05$), respectively. What's more, two interaction terms of physical activity and exercise type (i.e., PE × W1 and PE × W2) could all significantly predict MPA. In addition, the 95% CI of them did not include zero, which means that exercise type can effectively moderate the correlation between physical activity and MPA. Thus, in order to better analyze and explore the potential moderating effect of exercise type, a simple slope testing was performed, and the plot of the relation between physical activity and MPA at different kinds of exercise type was also clearly described in Fig. 1.

Simple slope analyses revealed that as for separated net exercise ($\beta = -0.132$) and confrontation exercise ($\beta = -0.009$), physical activity was positively associated with

Table 4. The moderating effect of exercise type between physical activity and MPA

Outcome	Predictors	β	t	Lower CI	Upper CI
MPA	Gender	0.271	2.41*	0.050	0.491
	Major	−0.169	−1.99*	−0.335	−0.002
	PA	−0.266	−3.85***	−0.401	−0.130
	W1	0.263	2.04*	0.010	0.516
	W2	0.445	2.44*	0.087	0.802
	W3	0.059	0.55	−0.153	0.272
	PA × W1	0.397	2.92**	0.131	0.664
	PA × W2	0.276	2.22*	0.032	0.519
	PA × W3	0.202	1.97	−0.001	0.405
Model fit index	R^2	0.064			
	$F_{(9.598)}$	4.577***			

Note: MPA, mobile phone addiction; PA, physical activity; the reference group, aerobic endurance programs (n = 364); W1, separated net programs (n = 63); W2, confrontation programs (n = 70); W3, difficulty beauty programs (n = 111); Lower CI, lower-level confidence interval; Upper CI, upper-level confidence interval; β = standardized coefficient.* $P < 0.05$; ** $P < 0.01$; *** $P < 0.001$.

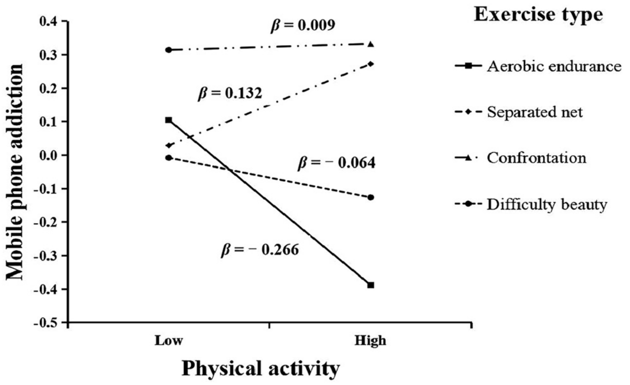

Note: Aerobic endurance programs (n=364); Separated net programs (n=63); Confrontation programs (n=70); Difficulty beauty programs (n=111); β = standardized coefficient

Fig. 1. The plot of the relationship between PA and MPA at different kinds of exercise type

Table 3. Pearson's correlation between physical activity, MPA and its four dimensions (N = *608*)

Variables	1	2	3	4	5	6
1. Physical activity	——					
2.Withdrawal symptoms	−0.113**	——				
3.Salience behavior	−0.035	0.711***	——			
4.Social comfort	−0.125**	0.562***	0.503***	——		
5.Mood changes	−0.087*	0.641***	0.635***	0.428***	——	
6.Mobile phone addiction	−0.109**	0.914***	0.863***	0.734***	0.789***	——

Note: * $P < 0.05$; ** $P < 0.01$; *** $P < 0.001$.

Unauthenticated | Downloaded 02/19/24 07:20 AM UTC

MPA, but that was not significant ($P > 0.05$). In contrast, in terms of difficulty beauty exercise and aerobic endurance exercise, physical activity was negatively associated with MPA, respectively. More important, although the correlation in difficulty beauty exercise was not significant ($\beta = -0.064$, $P > 0.05$), but that was highly significant in aerobic endurance exercise ($\beta = -0.266$, $P < 0.001$). These results may be indicative that actively engaging in aerobic endurance exercise would be greatly beneficial to reduce MPA for undergraduate students.

DISCUSSIONS

In recent years, the influence of physical activity on MPA has begun to greatly attract people's attention, especially in areas of behavioral addiction and public health. Therefore, a growing body of empirical research (e.g., survey and experimental study) has dedicated to examine the relationship between them, and so far, some critical findings have also been found. However, the potential effects (e.g., mediation, moderation, and moderated mediation) underlying this association remains largely unknown by now. Consequently, through the sample from Chinese undergraduates, the present study was to examine the correlation between physical activity and MPA again, and to testify whether the exercise type played a moderating role underlying this relation. The results suggested that physical activity was negatively associated with MPA, and with the enhancement of physical activity level, the score of MPA displayed an obvious decreasing tendency. While activity level increasing from none exercise to medium exercise, there was an optimum dose-response relationship between physical activity and MPA. Moreover, the correlation between them was moderated by exercise type, especially in aerobic endurance exercise; that is, the higher level of physical activity the undergraduates performed, the lower degree of MPA they would suffer. As follows, the principal findings in the current study would be specially examined one by one.

This study indicated that gender may be a critical demographic indicator to predict MPA, because the frequency and percentages of possible MPA and MPA for female college students were higher than no MPA, which is congruent with previous research (Demirci et al., 2015; Jiang & Zhao, 2016). Furthermore, according to prior work (Yang et al., 2019, 2020), that for undergraduates from liberal arts were also significantly higher than those from science majors, which suggested that major was also a very imperative predictor. Actually, the main causes about university students' difference in major were largely ascribed to the distinction between gender of them. As an example, some previous studies disclosed that female would be more likely to establish and maintain sociable relationships by mobile phones (Demirci et al., 2015; Rees & Noyes, 2007). Jiang and Zhao (2016) revealed that compared with their counterparts, the female often went shopping online and preferred to communicate with others through those advanced electronic devices. Billieux (2012) summarized published studies and pointed out that females may usually have more intensive emotional involvement, so they were more likely to experience dependence on mobile phones than males did. Additionally, that about 8% MPA among undergraduates in China obtained from the current study was consistent with an existing result reported by Mei et al. (2018).

As prior research suggested (Barkley & Lepp, 2016; Leep et al., 2013; Yang et al., 2019), the current study manifested that physical activity was reversely correlated with MPA, suggesting that physical activity might be a protective factor against individuals' MPA. Xiang et al. (2020) did find that MPA was negatively related to sedentary behavior; Fountaine, Liguori, Mozumdar, and Schuma (2011) and Leep et al. (2013) also argued that sedentary behavior may decrease physical activity in a sense. Accordingly, sedentary behavior may be an interfering factor between physical activity and MPA; that is, MPA could reduce university students' physical activity by increasing the probability of sedentary behaviors. In addition, Rebold, Sheehan, Dirlam, Maldonado, and O'Donnell (2016) disclosed that students who used various sport apps on mobile phones during physical exercise could decrease their desire for participating in vigorous-intensity physical exercise. However, they also put forward that listening to a little agreeable music using mobile phones while physical exercise, may also enhance exercise intensity (Rebold, Andrew, Sanders, Barkley, & Rosaria, 2015). These findings are inclined to agree with the notion that mobile phones may be a double-edged sword, and the influence of them on physical activity may largely rely on that how to properly treat and use them in daily lifestyle, which is fairly verified by a latest review about the role of mobile phones on physical activity promotion (Feter, Dos Santos, Caputo, & da Silva, 2019).

The third paramount finding of the current study was that with the increase of physical activity level, the score of MPA presented an evident tendency of decreasing, as well as its four dimensions, especially the level from none exercise to medium exercise. However, this positive effect was hardly found from medium exercise to high exercise. These results may imply that physical activity can be viewed as a practical intervening means to cope with MPA, but an indispensable precondition may be that exercise volume should reach to medium exercise level. Hence, it was not difficult to find that there was an optimum dose-response relationship in the course of reducing MPA by physical activity. In reality, except for those individuals who are trained before, the high exercise level may provide the general population with some passive effects with a large extent. According to the findings reported by Wang and Zhou (2012), compared with the low-intensity or high-intensity exercise groups, moderate-intensity aerobic exercise can produce the most optimum dose-response for university students' inhibition control. Furthermore, in a meta-analytical investigation of acute exercise on speed and accuracy with cognition, Mcmorris and Hale (2012) also stated that moderate-intensity exercise showed a significantly larger mean effect size than those in low-intensity or high-intensity exercise.

Unauthenticated | Downloaded 02/19/24 07:20 AM UTC

As we all know, the classical law of Yerkes-Dodson (i.e., rule of inverted U curve) implies that the relationship between motive intensity and working efficiency may be an inverted U curve rather than a linear relation, and insufficient or high-intensity motive may not be conducive to the improvement of working efficiency; therefore, moderate-intensity motive could be the most efficient level to perform missions (Yerkes & Dodson, 1908). Based on these above, it is easy for us to detect that as for college students, medium exercise level could be an effective and practical exercise standard to apply to the intervention in MPA.

The finding of the utmost significance in the present study was that exercise type can moderate the relationship between physical activity and MPA. Especially for the aerobic endurance exercise, the enhancement of activity level can largely bring us more effective attenuation of MPA. That is, the higher level of physical activity the undergraduates conducted, the lower degree of MPA they would suffer. Even though, there was no statistically significant difference among three groups of MPA above, but it can be still seen that compared with three other exercise types, the proportion of possible MPA and MPA in aerobic endurance exercise was the lowest, and that of no MPA was also the highest, which would be viewed as an essential indirect evidence to support this finding. Furthermore, Zhu (2017) conducts an experimental research and find that after exercise intervention with 8 weeks by programs including running and jogging, which can be ascribed to aerobic endurance exercise, some positive behavioral changes for college students addicted to mobile phones had been obviously produced in final, such as improving withdrawal symptoms, decreasing salient behaviors, alleviating social comfort, ameliorating mood changes, and the obvious reduction of the total scores of mobile phone addiction for them. Simultaneously, the same research results can be also acquired from an another study conducted by Bu (2014). However, this result may be contradicted in part with findings from Xu (2019), who found that in terms of addressing MPA, aerobic fitness exercise can produce the significantly positive effects to university students, but the intervention effects from other exercise programs (e.g., basketball and badminton) may be more effective than that one. One possible explanation is that sample sizes of the experimental groups are too small, because each group only contains 7 to 8 subjects so that the results might be unreliable or inaccurate. Another potential reason is that the control of the experimental process is not strict and serious, especially the aerobic fitness group may use sport apps on mobile phones during physical exercise so that the treatment effect of them may be largely affected at last.

The reason that the effect of aerobic endurance exercise was the best can be reasonably explained from the perspective of motor skills, proposed firstly by Bolton in 1957, an exercise psychologist from USA (Schmidt & Lee, 1998, pp. 10–16; Schmidt & Wrisberg, 2000, pp. 181–186). In accordance with the primary standpoint about that, confrontation, separated net and part difficult beauty programs can be classified as an exercise of open motor skills, in contrary the majority of aerobic endurance programs can be classified as an exercise of closed motor skills. Compared with the latter, open motor skills are frequently more complex and difficult to implement, because those involve more contextual factors during physical exercise. In addition, exercise effect of open motor skills may not only depend on exercise intensity, duration and frequency, but also the final consequence (i.e., victory or defeat), which would be the most inevitable disturbing or risky factor (Chai, He, & Jiang, 2010; Wang & Li, 2007). Consequently, exercise effect from closed motor skills might usually be more satisfactory than its counterpart, which is congruent with previous intervention effects on undergraduates' smartphone addiction. Liu, Xiao, et al. (2019) conducted a meta-analysis and put forward that students participating in closed motor skills displayed significant intervention effect on decreasing MPA, but those engaging in open motor skills may not acquire more satisfactory intervention effects.

Billieux et al. (2015) did firmly state that problematic mobile phone use may not be classified as a behavioral addiction, but an increasing number of published studies frown on this and intensely propose that MPA can be labeled as an addiction behavior (Chóliz, 2010; Kim, Lee, Lee, Nam, & Chung, 2014; Kwon et al., 2013; Lin et al., 2014; Liu, Xiao, et al., 2019; Robert, 2006; Wang, Sigerson, Jiang, & Cheng, 2018). It is widely admitted that, as a kind of behavioral addiction, mobile phone users usually show a series of physiological or psychological symptoms similar to substance dependence. Whereas, it is fortunate that those can be greatly alleviated or eliminated by physical activity, in particular the regular and continuous aerobic exercise. Specifically speaking, physical activity can attenuate anxiety and depression disorders (Carek, Laibstain, & Carek, 2011; Wegner et al., 2014), improving sleep quality and decreasing sense of loneliness (Hedlund, Villard, Lundell, & Sjöberg, 2019; Smith, Banting, Eime, O'Sullivan, & Uffelen, 2017); alleviating stress in daily life and enhancing life satisfaction degree (Gellert, Wienert, Ziegelmann, & Kuhlmey, 2019; Hearing et al., 2016; Park, 2014).

In addition, Kim (2013) generalized and discussed the applicability of exercise rehabilitation for smartphone addiction, and pointed out that physical exercise would be beneficial to address this troublesome addiction problem from both physiological and psychological aspects. A latest meta-analysis also suggested that physical exercise could be a paramount approach to intervene in MPA, and longer intervention duration may bring greater intervention effects (Liu, Xiao, et al., 2019). Simultaneously, physical exercise could decease some unnecessary sedentary activities and redundant screen time spending on mobile phones (Barkley & Lepp, 2016; Lepp et al., 2013; Penglee et al., 2019), which would be also a potential path to attenuate MPA.

LIMITATIONS AND IMPLICATIONS

This study made some important contributions, but there were still a few limitations we need to attach much

Unauthenticated | Downloaded 02/19/24 07:20 AM UTC

importance. At first, the data were collected totally relying on self-report by subjects, so the final results may be affected to some extent. Secondly, a cross-sectional survey study cannot get some casual inference. Thus, the longitudinal or experimental study should be carried out to obtain more persuasive consequences. Thirdly, sample size in the current study was not considerable and quota sampling did not belong to the method of probability sampling, so the results may not be effectively generalized to other groups. Fourthly, the internal consistency of PARS-3 was 0.64, which might be largely affected by sample sizes and only three items included in itself. Fifthly, with respect to evaluate the physical activity, many modern equipment of artificial intelligence (e.g., 3D-sensor pedometer, ActiGraph accelerometer, sport bracelets and heart rate band) can be also employed afterward. In addition, apart from the moderating effect conducted by this study, more potential effects under this correlation can be further screened in coming days. Just as sedentary behaviors and screen time we mentioned above, might be also a mediator or moderator between physical activity and MPA. Last but not least, due to the quota sampling is not a means of probability random sampling and the condition for overall composition of sampling frame is that the proportion of females are relatively high, this study may exist some distribution differences in gender.

Regardless of several limitations mentioned above, the present study reaped lots of valuable fruits at the same time. First and foremost, this study extended the previous work and further demonstrated the negative relationship between physical activity and MPA. More important, the current study may be the first one to try to examine the potential moderating effects underlying this association, and surprisingly found that exercise type played an indispensable moderating role between physical activity and MPA. Thirdly, there was also an optimum dose-response relation between them, and the medium activity level may bring the most rewarding intervention effect for college students addicted to mobile phones. As for them, a practicable exercise prescription would be that taking at least three times per week, and exercise duration lasting for 30–60 min with moderate-intensity physical activity, which is congruent with recommendations of physical activity for the general population reported by WHO (2007). However, which kind of exercise type should be optimally embraced for undergraduates to attenuate MPA, it is no doubt that the present study has given us an effective and reliable response. Last but not least, that actively engaging in aerobic endurance exercise such as running, swimming, riding bicycle, and jogging with the medium activity level, would be the best exercise strategy to treat this troublesome addiction problem for them in daily lifestyle behavior. In summary, everyone should bear in mind a truth that exercise is medicine.

CONCLUSIONS

The present study may be the first one to testify whether the exercise type could play a moderating role underlying the correlation between physical activity and MPA. As expected, physical activity could be a critical protective factor against MPA. There was an optimum dose-response relation during physical activity decreasing MPA, and the medium activity level would produce the most rewarding intervention effect to attenuate MPA. Regarding aerobic endurance exercise, the higher level of physical activity the undergraduates implemented, the lower degree of MPA they would undergo. It is no doubt that physical activity especially the aerobic endurance exercise with medium activity level, would be a practicable exercise strategy in daily lifestyle behavior to deal with this severe addiction problem for undergraduates in China or even in other countries all over the world.

Funding sources: This study was supported by the Innovation Project of Graduate School of South China Normal University (2018LKXM011).

Authors' contributions: G.Y. and Y.X.L. designed this study. The funding acquisition was made by G.Y. G.Y. and Y.X.L. collected and analyzed the data. G.Y. and S.J.L. discussed the results. The original manuscript was drafted by G.Y., and the revision was examined by C.N.L., G.J. and S.T.W. At last, all authors confirmed the final submitted version.

Conflicts of interest: The authors declare that the research was conducted in the absence of any commercial or financial relationships that could be constructed as a potential conflict of interest.

Acknowledgement: The authors are grateful to all the participants who were involved in this study.

REFERENCES

Aleksandar, V., Vladica, V., Dusan, S., Miodrag, S., Kristijan, M., Miodrag, S., . . . Olivera, R. (2018). Relationship between the manner of mobile phone use and depression, anxiety, and stress in university students. *International Journal of Environmental Research and Public Health*, *15*(4), 697–711. https://doi.org/10.3390/ijerph15040697.

Barkley, J. E., & Lepp, A. (2016). Mobile phone use among college students is a sedentary leisure behavior which may interfere with exercise. *Computers in Human Behavior*, *56*, 29–33. https://doi.org/10.1016/j.chb.2015.11.001.

Bianchi, A., & Phillips, J. G. (2005). Psychological predictors of problem mobile phone use. *Cyberpsychology Behavior*, *8*(1), 39–51. https://doi.org/10.1089/cpb.2005.8.39.

Billieux, J. (2012). Problematic use of the mobile phone: A literature review and a pathways model. *Current Psychiatry Reviews*, *8*(4), 299–307. https://doi.org/10.2174/157340012803520522.

Billieux, J., Maurage, P., Lopez-Fernandez, O., Kuss, D. J., & Griffiths, M. D. (2015). Can disordered mobile phone use be considered a behavioral addiction? An update on current evidence and a comprehensive model for future research.

Unauthenticated | Downloaded 02/19/24 07:20 AM UTC

Current Addiction Reports, *2*(2), 156–162. https://doi.org/10.1007/s40429-015-0054-y.

Bu, Y. L. (2014). Effect of physical exercise on college students' mobile phone addiction. *Modern Preventive Medicine*, *41*(7), 1242–1245.

Carek, P. J., Laibstain, S. E., & Carek, S. M. (2011). Exercise for the treatment of depression and anxiety. *International Journal of Psychiatry Medicine*, *41*, 15–28. https://doi.org/10.2190/PM.41.1.c.

Chai, J., He, J. P., & Jiang, L. J. (2010). Principle of open style sports skill learning and its application in basketball teaching. *Journal of Physical Education*, *17*(5), 65–68. https://doi.org/10.16237/j.cnki.cn44-1404/g8.2010.09.015.

Chen, L., Zheng, Y., Tang, W. .J., Yang, F. Y., Xie, X. D., & He, J. C. (2016). Mobile phone addiction levels and negative emotions among Chinese young adults: The mediating role of interpersonal problems. *Computers in Human Behavior*, *55*, 856–866. https://doi.org/10.1016/j.chb.2015.10.030.

Chóliz, M. (2010). Mobile phone addiction: A point of issue. *Addiction*, *105*(2), 373–374. https://doi.org/10.1111/j.1360-0443.2009.02854.x.

CNNIC (2019). China Internet Network Information Center. The 44th Statistics Report the Development of China Internet Network. Available at: http://www.199it.com/archives/930922.html [Accessed 25 Jan 2020].

Cohen, J. (1988). *Statistical power analysis for the behavioral sciences* (2nd.). Hillsdale, New Jersey: Lawrence Erlbaum Associates.

Demirci, K., Akgönül, M., & Akpinar, A. (2015). Relationship of smartphone use severity with sleep quality, depression, and anxiety in university students. *Journal of Behavioral Addictions*, *4*(2), 85–92. https://doi.org/10.1556/2006.4.2015.010.

Fan, R. C., & Yuan, X. Y. (2015). Experimental research on sports intervention on college students' mobile addiction. *Journal of Contemprary Sports Science*, *5*(20), 1–2.

Feter, N., Dos Santos, T. S., Caputo, E. L., & da Silva, M. C. (2019). What is the role of smartphones on physical activity promotion? A systematic review and meta-analysis. *International Journal of Public Health*, *64*(5), 1210–1216. https://doi.org/10.1007/s00038-019-01210-7.

Fountaine, C. J., Liguori, G. A., Mozumdar, A., & Schuma, J. M. (2011). Physical activity and screen time, sedentary behaviors in college students. *International Journal of Exercise Science*, *4*(13), 102–112.

Gellert, P., Wienert, J., Ziegelmann, J. P., & Kuhlmey, A. (2019). Profiles of physical activity biographies in relation to life and aging satisfaction in older adults: Longitudinal findings. *European Review of Aging and Physical Activity*, *16*(1), 221–226. https://doi.org/10.1186/s11556-019-0221-6.

Ge, R. Z., Zhong, X. M., & Chen, R. (2015). Research on the influence of sports intervention on college students' mobile phone dependence. *Modern Preventive Medicine*, *42*(21), 3919–3921.

Haruka, T., Tomoko, N., Akiyo, T., & Hisataka, S. (2017). Association between excessive use of mobile phone and insomnia and depression among Japanese adolescents. *International Journal of Environmental Research and Public Health*, *14*(7), 701–714. https://doi.org/10.3390/ijerph14070701.

Hayes, A. F. (2013). *Introduction to mediation, moderation, and conditional process analysis: A regression-based approach*. New York, NY: Guilford Press.

Hearing, C. M., Chang, W. C., Szuhany, K. L., Deckersbach, T., Nierenberg, A. A., & Sylvia, L. G. (2016). Physical exercise for treatment of mood disorders: A critical review. *Current Behavioral Neuroscience Reports*, *3*(4), 350–359. https://doi.org/10.1007/s40473-016-0089-y.

Hedlund, E. R., Villard, L., Lundell, B., & Sjöberg, G. (2019). Physical exercise may improve sleep quality in children and adolescents with Fontan circulation. *Cardiology in the Young*, *29*(7), 136–148. https://doi.org/10.1017/s1047951119001136.

Jiang, Z. C., & Zhao, X. X. (2016). Self-control and problematic mobile phone use in Chinese college students: The mediating role of mobile phone use patterns. *BMC Psychiatry*, *16*(1), 416–423. https://doi.org/10.1186/s12888-016-1131-z.

Kim, H. (2013). Exercise rehabilitation for smartphone addiction. *Journal of Exercise Rehabilitation*, *9*(6), 500–505. https://doi.org/10.12965/jer.130080.

Kim, S. E., Kim, J. W., & Jee, Y. S. (2015). Relationship between smartphone addiction and physical activity in Chinese international students in Korea. *Journal of Behavioral Addictions*, *4*(3), 200–205. https://doi.org/10.1556/2006.4.2015.028.

Jun, S. (2016). The recipocal logitudinal relationships between mobile phone addiction and depressive symptoms among Korean adolescents[J]. *Computers in Human Behavior*, *58*, 179–186. https://doi:10.1016/j.chb.2015.12.061.

Kim, D., Lee, Y., Lee, J., Nam, J. K., & Chung, Y. (2014). Development of korean smartphone addiction proneness scale for youth. *PloS One*, *9*(5), e97920. https://doi.org/10.1371/journal.pone.0097920.

Kwon, M., Lee, J. Y., Won, W. Y., Park, J. W., Min, J. A., & Hahn, C., ... Kim, D. J. (2013). Development and validation of a smartphone addiction scale (SAS). *PloS One*, *8*(2), e56936. https://doi.org/10.1371/journal.pone.0056936.

Leep, A., Barkley, J. E., & Karpinski, A. C. (2015). The relationship between cell phone use and academic performance in a sample of U.S. college students. *Sage Open*, *5*(1), 1–9. https://doi.org/10.1177/2158244015573169.

Lepp, A., Barkley, J. E., Sanders, J. G., Rebold, M., & Gates, P. (2013). The relationship between cell phone use, physical and sedentary activity, and cardiorespiratory fitness in a sample of U.S. college students. *International Journal of Behavioral Nutrition & Physical Activity*, *10*(1), 79–87. https://doi.org/10.1186/1479-5868-10-79.

Li, J., Lepp, A., & Barkley, J. E. (2015). Locus of control and cell phone use: Implications for sleep quality, academic performance, and subjective well-being. *Computers in Human Behavior*, *52*, 450–457. https://doi.org/10.1016/j.chb.2015.06.021.

Liang, D. Q., & Liu, S. J. (1994). The relationship between stress level and physical exercise for college students. *Chinese Mental Health Journal*, *8*(1), 5–6.

Lin, Y.-H., Chang, L.-R., Lee, Y.-H., Tseng, H.-W., Kuo, T. B. J., & Chen, S.-H. (2014). Development and validation of the smartphone addiction inventory (SPAI). *PloS One*, *9*(6), e98312. https://doi.org/10.1371/journal.pone.0098312.

Liu, X., Liu, X., & Wei, R. (2014). Maintaining social connectedness in a fast-changing world: Examining the effects of mobile phone uses on loneliness among teens in Tibet. *Mobile Media & Communication*, *2*(3), 318–334. https://doi.org/10.1177/2050157914535390.

Unauthenticated | Downloaded 02/19/24 07:20 AM UTC

Liu, S., Xiao, T., Yang, L., & Loprinzi, P. D. (2019). Exercise as an alternative approach for treating smartphone addiction: A systematic review and meta-analysis of random controlled trials. *International Journal of Environmental Research and Public Health*, *16*(20), 3912–3927. https://doi.org/10.3390/ijerph16203912.

Mcmorris, T., & Hale, B. J. (2012). Differential effects of differing intensities of acute exercise on speed and accuracy of cognition: A meta-analytical investigation. *Brain and Cognition*, *80*(3), 338–351. https://doi.org/10.1016/j.bandc.2012.09.001.

Mei, S. L., Chai, J. X., Wang, S. B., Ng, C. H., Ungvari, G. S., & Xiang, Y. T. (2018). Mobile phone dependence, social support and impulsivity in Chinese university students. *International Journal of Environmental Research and Public Health*, *15*(3), 504–510. https://doi.org/10.3390/ijerph15030504.

Park, S. (2014). Associations of physical activity with sleep satisfaction, perceived stress, and problematic Internet use in Korean adolescents. *BMC Public Health*, *14*(1), 1143–1148. https://doi.org/10.1186/1471-2458-14-1143.

Penglee, N., Christiana, R. W., Battista, R. A., & Rosenberg, E. (2019). Smartphone use and physical activity among college students in health science-related majors in the United States and Thailand. *International Journal of Environmental Research and Public Health*, *16*(8), 1315–1323. https://doi.org/10.3390/ijerph16081315.

Rebold, M. J., Andrew, L., Sanders, G. J., Barkley, J. E., & Rosaria, S. M. (2015). The impact of cell phone use on the intensity and liking of a bout of treadmill exercise. *PloS One*, *10*(5), e0125029. https://doi.org/10.1371/journal.pone.0125029.

Rebold, M. J., Sheehan, T., Dirlam, M., Maldonado, T., & O'Donnell, D. (2016). The impact of cell phone texting on the amount of time spent exercising at different intensities. *Computers in Human Behavior*, *55*, 167–171. https://doi.org/10.1016/j.chb.2015.09.005.

Rees, H., & Noyes, J. M. (2007). Mobile telephones, computers, and the Internet: Sex differences in adolescents' use and attitudes. *Cyberpsychology & Behavior*, *10*(3), 482–484. https://doi.org/10.1089/cpb.2006.9927.

Rhodes, R. E., Janssen, I., Bredin, S. S. D., Warburton, D. E. R., & Bauman, A. (2017). Physical activity, health impact, prevalence, correlates and interventions. *Psychology & Health*, *32*(8), 942–975. https://doi.org/10.1080/08870446.2017.1325486.

Robert, W. (2006). *Theory of addiction*. US: Springer Press, Wiley-Blackwell.

Schmidt, R. A., & Lee, T. D. (1998). *Motor control and learning: A behavior emphasis*. Champaign. IL: Human Kinetics.

Schmidt, R. A., & Wrisberg (2000). *Motor- learning and performance*. Champaign. IL: Human Kinetics.

Servick, K. (2015). Mind the phone. *Science*, *350*(6266), 1306–1309. https://doi.org/10.1126/science.350.6266.1306.

Sheng, J. G., Jiang, Y. C., & Gao, S. Q. (2018). A study on the effect of physical exercise on coping self-efficacy and coping style of college students. *Journal of Sports Sciences*, *39*(3), 30–37. https://doi.org/10.13598/j.issn1004-4590.2018.03.006.

Smith, G. L., Banting, L., Eime, R., O'Sullivan, G., & Uffelen, J. G. Z. V. (2017). The association between social support and physical activity in older adults: A systematic review. *International Journal of Behavioral Nutrition & Physical Activity*, *14*(1), 56–63. https://doi.org/10.1186/s12966-017-0509-8.

Wang, K. (2016). The influence of sports intervention on the degree of college students' mobile addiction. *Sports Research and Education*, *8*(3), 109–112. https://doi.org/10.16207/j.cnki.2095-235x.2016.03.023.

Wang, J., & Li, Z. H. (2007). Comparison on instruction methods of motor skills between open-loop and closed-loop in the women students of university. *Journal of Tianjin University of Sport*, *27*(1), 67–73. https://doi.org/10.13297/j.cnki.issn1005-0000.2007.01.016.

Toda, M., Monden, K., Kubo, K., & Morimoto, K. (2006). Mobile phone dependence and health-related lifestyle of university students. *Social Behavior and Personality*, *34*(10), 1277–1284. https://doi.org/10.2224/sbp.2006.34.10.1277.

Wang, H.-Y., Sigerson, L., Jiang, H., & Cheng, C. (2018). Psychometric properties and factor structures of Chinese smartphone addiction inventory: Test of two models. *Frontiers in Psychology*, *9*, 1411–1421. https://doi.org/10.3389/fpsyg.2018.01411.

Wang, X. T., & Zhang, C. (2016). Research on the exercise intervention of mental health level of mobile phone addiction medical students. *China Higher Medicine Education*, *7*(9), 29–30. https://doi.org/10. 3969/j. issn.1002-1701.2016. 07.011.

Wang, Y. Y., & Zhou, C. L. (2012). The dose-response relationship between acute exercise intensity and inhibition control—case from ERP studies. *China Sport Science and Technology*, *34*(11), 42–49. https://doi.org/10.16469/j.css.2014.11.001.

Wegner, M., Helmich, I., Machado, S., Nardi, A., Arias-Carrion, O., & Budde, H. (2014). Effects of exercise on anxiety and depression disorders: Review of meta-analyses and neurobiological mechanisms. *CNS & Neurological Disorders Drug Targets*, *13*(6), 1002–1014. https://doi.org/10.2174/1871527313666140612102841.

WHO (2007). *The world health report 2007-a safer future: Global public health security in the 21st century*. Geneva, Switzerland: World Health Organization.

WHO (2010). *Global recommendations of physical activity for health*. Geneva, Switzerland: World Health Organization.

Xavier, C., Chamarro, A., Ursula, O., Beatriz, R., & Mariona, P. (2018). Problematic use of the internet and smartphones in university students: 2006–2017. *International Journal of Environmental Research and Public Health*, *15*(3), 475–487. https://doi.org/10.3390/ijerph15030475.

Xia, X. W., Huang, J. L., & Liu, D. (2018). Investigation and study of university graduate students' physical exercise behavior affecting factors. *Journal of Physical Education*, *25*(5), 102–108. https://doi.org/10.16237/j.cnki.cn44-1404/g8.2018.05.010.

Xiang, M. Q., Lin, L., Wang, Z. R., Li, J., Xu, Z. B., & Hu, M. (2020). Sedentary behavior and problematic smartphone use in Chinese adolescents: The moderating role of self-control. *Frontiers in Psychology*, *10*, 3032–3040. https://doi.org/10.3389/fpsyg.2019.03032.

Xiong, J., Zhou, Z. K., Chen, W., You, Z. Q., & Zhai, Z. Y. (2012). Development of the mobile phone addiction tendency scale for college students. *Chinese Mental Health Journal*, *26*(3), 222–225. https://doi.org/10.3969/j.issn.1000-6729.2012.03.013.

Xu, X. L. (2019). Effects of physical exercise on college students' mobile phone dependence. *Hubei Sports Science*, *38*(1), 57–60.

Unauthenticated | Downloaded 02/19/24 07:20 AM UTC

Yang, G., Li, Y. X., Liu, H. Y., & Wang, S. T. (2020). Analysis of the relationship between physical exercise and cellphone dependence of university students in Guangzhou. *Journal of Physical Education*, *27*(1), 117–125. https://doi.org/10.16237/j.cnki.cn44-1404/g8.20191003.005.

Yang, G., Tan, G. X., Li, Y. X., Liu, H. Y., & Wang, S. T. (2019). Physical exercise decreases the mobile phone dependence of university students in China: The mediating role of self-control. *International Journal of Environmental Research and Public Health*, *16*(21), 4098–4108. https://doi.org/10.3390/ijerph16214098.

Yerkes, R. M., & Dodson, J. D. (1908). The relation of strength of stimulus to rapidity of habit-formation. *Journal of Comparative Neurology and Psychology*, *18*, 459–482. https://doi.org/10.1002/cne.920180503.

Young, K. S. (1998). Internet addiction: The emergence of a new clinical disorder. *Cyberpsychology & Behavior*, *1*, 237–244. https://doi.org/10.1089/cpb.1998.1.237.

Zagalaz-Sánchez, M. L., Cachón-Zagalaz, J., Sánchez-Zafra, M., & Lara-Sánchez, A. (2019). Mini review of the use of the mobile phone and its repercussion in the deficit of physical activity. *Frontiers in Psychology*, *10*, 1307–1312. https://doi.org/10.3389/fpsyg.2019.01307.

Zhang, J. S., Hu, R. K., & Xu, Y. Q. (2016). Research on the intervention of college students' mobile phone dependence by outdoor development movement. *Science Technology Visual*, *10*, 321–326. https://doi.org/10.19694/j.cnki.issn2095-2457.2016.09.237.

Zhu, Y. F. (2017). Intervention study of physical exercise on college students' mobile addiction tendency. *Zhejiang Sports Science*, *6*(5), 90–97.

Open Access. This is an open-access article distributed under the terms of the Creative Commons Attribution-NonCommercial 4.0 International License (https://creativecommons.org/licenses/by-nc/4.0/), which permits unrestricted use, distribution, and reproduction in any medium for non-commercial purposes, provided the original author and source are credited, a link to the CC License is provided, and changes – if any – are indicated.

Unauthenticated | Downloaded 02/19/24 07:20 AM UTC
